# A severity comparison of leukoaraiosis in ischemic and hemorrhagic stroke: a retrospective study

**DOI:** 10.3389/fneur.2024.1425440

**Published:** 2024-10-28

**Authors:** Bendik Søfteland, Nedim Leto, Halvor Næss

**Affiliations:** ^1^Department of Clinical Medicine, Faculty of Medicine, University of Bergen, Bergen, Norway; ^2^Department of Neurology, Haukeland University Hospital, Bergen, Norway

**Keywords:** leukoaraiosis, stroke pathophysiology, ischemic stroke, hemorrhagic stroke, stroke outcome improvements

## Abstract

**Introduction:**

Leukoaraiosis (LA) is a common neuroradiological finding in patients suffering from stroke. Cerebral small-vessel disease (SVD) is one of the primary causes of both ischemic stroke and hemorrhagic stroke (intracerebral hemorrhage, ICH) and the development of LA. Significant evidence that LA predicts the risk of functional dependency and mortality exists. However, studies examining the difference in LA severity between ischemic stroke and hemorrhagic stroke are lacking. We therefore aimed to compare the severity and abundance of LA in the two stroke subgroups.

**Methods:**

All patients admitted to the Department of Neurology, Haukeland University Hospital, with an ischemic stroke and intracerebral hemorrhagic (ICH) stroke diagnosed between 2006 and 2020 were included in the study. We collected patient data on risk factors and clinical and radiological findings and outcomes from our local stroke registry. The presence and severity of LA were assessed using the Fazekas score based on CT imaging. We evaluated the outcome using the modified Rankin Score (mRS) 7 days post-stroke.

**Results:**

A total of 5,084 patients were included in our analyses: 4437 (87%) with ischemic stroke and 647 (13%) with ICH. LA was present in 2476 (45%) patients. In our ordinal logistic regression model, adjusting for age, sex, known hypertension, known diabetes mellitus, and smoking, LA was more severe and more abundant in ICH patients compared to ischemic stroke patients (Fazekas score: 1, OR: 1.54; Fazekas score: 2, OR: 1.88; and Fazekas score: 3, OR 2.13; *p* < 0.001). Increasing severity of LA was associated with worse functional outcomes in both groups (ischemic stroke, OR: 1.49; *p* < 0.001 and ICH, OR: 1.36; *p* < 0.025).

**Conclusion:**

In this study, LA was more severe and abundant in patients with ICH.

## 1 Introduction

Leukoaraiosis (LA) is a common neuroradiological finding in patients suffering from stroke. Cerebral small-vessel disease (SVD) is one of the primary causes of stroke and the development of LA (1). SVD is a common neurovascular disease process that can cause significant impairment both in stroke and dementia (2, 3).

The pathophysiology of LA is not known. Typically, LA is caused by chronic hypoperfusion of subcortical white matter, leading to impairment of brain reserve capacity to injury (2). Cerebral amyloid angiopathy, hypertension, and increasing age are all risk factors for developing LA (4–6).

LA is an independent predictor of post-stroke functional dependency and mortality, both in ICH and ischemic stroke, making health intervention against SVD development an important target (7, 8). Differences in mortality rates between ICH and ischemic stroke exist. This has primarily been related to higher stroke severity and the absence of effective treatment in ICH (9). However, studies investigating differences in the severity of LA in the two stroke subtypes, and the impact this has on differences in patient outcomes, are lacking. Because LA is associated with cerebral amyloid angiopathy, hypertension causing SVD, and worse stroke outcomes, we hypothesized that moderate-to-severe LA is more frequent in ICH patients than in ischemic stroke patients.

## 2 Methods

### 2.1 Study population and design

We included all ICH and ischemic stroke patients admitted to Haukeland University Hospital, Bergen, Norway, between 2006 and 2020. Patient data were prospectively collected from our local stroke registry. We excluded traumatic ICH and hemorrhages due to intracranial aneurysms.

Data on risk factors and clinical and radiological findings and outcomes were collected. We recorded hypertension (known diagnosis), diabetes (known diagnosis), smoking (ever), atrial fibrillation (AF) (known diagnosis or documented at hospital stay), and coronary heart disease (known diagnosis or documented at hospital stay) as risk factors. The National Institute of Stroke Scale (NIHSS) was used to assess stroke severity at hospital admission. All patients underwent computed tomography (CT) imaging at hospital admission. We defined LA as CT hypoattenuation in the periventricular white matter area or in the deep white matter area. An experienced senior neurologist graded the severity of LA using the Fazekas score. Mild LA was defined as a Fazekas score of 1, moderate as a Fazekas score of 2, and severe as a Fazekas score of 3, in accordance with established definitions (10). The outcome was evaluated using the modified Rankin Score (mRS) after 7 days or at hospital discharge if earlier. We defined a mRS score of 0–2 as a good outcome and a mRS score of 3–6 as a poor outcome.

The study was approved by the local ethics committee.

### 2.2 Statistical analyses

We presented categorical variables as numbers (percentage) and continuous variables either presented as mean (standard deviation) or as median (interquartile range), depending on the normality distribution of data or not. We performed Pearson’s chi-squared test on selected categorical variables. Multivariate logistic regression was performed for comparison of abundance and severity of LA in ICH and ischemic stroke, adjusted for sex, age, known hypertension, known diabetes mellitus, and smoking. Separate multivariate logistic regression analyses were performed for outcome and severity of LA for both ICH stroke and ischemic stroke, adjusted for sex, age, and NIHSS score at admission. All analyses were performed using STATA version 17.0 (StataCorp 4905 Lakeway Drive, College Station, Texas 77845 USA).

## 3 Results

In total, 5,084 patients were included in our analyses: 4437 (87%) with ischemic stroke and 647 (13%) with ICH. LA was present in 2476 (45%) patients. LA was more abundant and severe in patients with ICH than in patients with ischemic stroke (*p* < 0.001). A comparison of the two stroke subgroups is presented in Table 1. Data regarding ischemic stroke etiology were available in 3582 (81%) patients, of which 924 (26%) were classified as lacunar stroke and 2658 (74%) as embolic stroke.

**Table 1:** Demographics of patients with ischemic stroke and ICH.

*N* = 5,084	Ischemic stroke	ICH
Male patients, *n* (%)	2,472 (55.7)	352 (54.4)
Female patients, *n* (%)	1,965 (44.3)	295 (45.6)
Age (years) at ictus, mean (SD^1^)	71.7 (31.0)	72.4 (14.0)
NIHSS^2^ at admission, mean (SD^1^)	5.6 (6.6)	11.4 (8.9)
Diabetes mellitus*, *n* (%)	699 (15.9)	82 (12.9)
Hypertension*, *n* (%)	2,378 (53.7)	330 (51.4)
Smoking^3^, *n* (%)	2,468 (59.3)	314 (58.0)
Prior ischemic stroke, *n* (%)	513 (11.7)	60 (9.4)
Prior hemorrhagic stroke, *n* (%)	45 (1.0)	34 (5.3)
Atrial fibrillation*, *n* (%)	871 (19.8)	124 (19.4)
Prior myocardial infarction, *n* (%)	624 (14.1)	72 (11.2)
Fazekas score CT, *n* (%)^4^		
0	2,531(57.0)	286 (44.2)
1	970 (21.9)	172 (26.6)
2	585 (13.2)	112 (17.3)
3	351 (7.9)	77 (11.9)

^1^Standard deviation. ^2^The National Institutes of Health Stroke Scale. ^3^Both former and active smokers. ^4^ Pearson’s chi2 (3) = 40.10, *p* < 0.001. ^*^Known diagnosis.

Unadjusted analyses showed that the association between ICH and LA increased by LA severity (Table 2). In adjusted analyses, we adjusted for sex, age, known hypertension, known diabetes mellitus, and smoking. When adjusting for these variables, the association was still present and increasing by LA severity (Table 2). Due to missing values for adjusting variables, 449 patients were excluded from the adjusted analyses. In comparison between ischemic embolic and lacunar strokes, the degree and presence of LA were lower in the embolic stroke group in adjusted analyses (Table 3).

**Table 2:** Multivariate logistic regression of stroke subtype (ischemic stroke and ICH) and severity of LA (Fazekas score CT).

	Unadjusted^a^				Adjusted^b^	
Fazekas score	OR	95% CI*	*p*-value	OR	95% CI*	*P*-value
0	1 (reference)			1 (reference)		
1	1.57	1.28–1.92	<0.001	1.54	1.23–1.94	<0.001
2	1.69	1.34–2.15	<0.001	1.88	1.45–2.44	<0.001
3	1.94	1.47–2.56	<0.001	2.13	1.56–2.89	<0.001

Reference is Fazekas score = 0. ^a^Number of observations = 5084. ^b^Number of observations = 4635. Adjusted for sex, age, known hypertension, known diabetes mellitus, and smoking (active and former). ^*^Confidence interval.

**Table 3:** Multivariate logistic regression of ischemic stroke etiology (embolic stroke and lacunar stroke) and severity of LA (Fazekas score CT).

	Unadjusted^a^				Adjusted^b^	
Fazekas score	OR	95% CI*	*P*-value	OR	95% CI*	*P*-value
0	1 (reference)			1 (reference)		
1	0.75	0.62–0.90	0.002	0.62	0.50–0.76	<0.001
2	0.81	0.62–1.02	0.071	0.63	0.49–0.82	<0.001
3	0.75	0.56–1.01	0.059	0.58	0.42–0.80	0.022

Reference is lacunar stroke = 0. ^a^Number of observations = 3582. ^b^Number of observations = 3380. Adjusted for sex, age, known hypertension, known diabetes mellitus, and smoking (active and former). ^*^Confidence interval.

In our outcome analysis model, presented in Table 4, increasing severity of LA was associated with poor outcome in both the ICH and the ischemic stroke groups. The OR values for patients with ICH and ischemic stroke were 1.36 (95% CI 1.04–1.78; *p* < 0.025) and 1.49 (95% CI 1.37–1.62; *p* < 0.001), respectively.

**Table 4:** Multivariate logistic regression with short-term outcome (mRS) as dependent variable.

	ICH^b^			Ischemic stroke^c^		
mRS score at 7 days/discharge	OR	95% CI^*^	*p*-value	OR	95% CI^*^	*p*-value
Fazekas score	1.36	1.04–1.78	<0.025	1.49	1.37–1.62	<0.001
Sex	1.07	0.66–1.76	<0.778	1.11	0.95–1.29	<0.175
Age	1.04	1.02–1.05	<0.001	1.04	1.03–1.05	<0.001
NIHSS^a^ score at admission	1.32	1.24–1.40	<0.001	1.29	1.26–1.31	<0.001

Adjusted for sex, age, and NIHSS^a^ score at admission. *Confidence interval. ^a^The National Institutes of Health Stroke Scale. ^b^Number of observations = 626. ^c^Number of observations = 4366.

## 4 Discussion

In accordance with our hypothesis, LA was more severe and more often present in patients suffering from ICH compared to ischemic stroke. This association was still present after adjusting for various risk factors. In the ischemic stroke group, the presence and degree of LA were lower in the embolic than in lacunar stroke. In our study, a high proportion of patients in the ICH and ischemic stroke groups had moderate-to-severe LA.

No studies have previously compared the severity and presence of LA between ICH and ischemic stroke. However, it is well-known that LA is a common finding in the elderly, especially among patients with ICH and ischemic stroke (6). Furthermore, LA is an independent risk factor for stroke recurrence and short-term and long-term mortality (8, 11, 12).

LA also increases stroke severity and worsens outcomes after a stroke. A Chinese study on patients with mild ICH reported that moderate-to-severe LA was associated with an increased NIHSS score at admission, and 3-month functional dependence was associated with increasing LA severity (13). An American study on LA severity and clinical outcome after ischemic stroke showed LA volume as an independent predictor of a higher 6-month mRS score in ischemic stroke patients (14). In our study, we saw that increasing LA severity is significantly associated with a worse mRS score at 7 days or at discharge.

The impact of LA on the risk of stroke recurrence varies by stroke subtype. In a study from 2019 including patients with an ischemic stroke, the hazard ratio for 1-year stroke recurrence in ICH patients with LA was 7.32 for ICH and 1.03 for ischemic stroke (12). Still, the absolute risk was higher for an ischemic stroke than for ICH (LA quartiles: 3.8, 4.5, 6.3, and 8.2% vs. 0.1, 0.4, 0.6, and 1.3%). The risk of stroke recurrence in both groups increased by the severity of LA (12).

The risk of mortality is also increased in stroke patients with LA. A Norwegian ICH study found an odds ratio of 1.6 for 30-day mortality and a hazard ratio of 1.6 for long-term mortality in patients with LA (8). For ischemic stroke, a study reported that mortality increases by an odds ratio of 1.87 in patients with prior asymptomatic lacunar stroke, which is associated with the presence of LA (15).

The strengths of this study are the high number of patients included and the prospective data collection, which reduced the risk for recall bias. Furthermore, extensive amounts of radiological, clinical, and background data were available in this local stroke registry, which gave us the possibility of adjusting for known confounders. The limitations of this study are the retrospective design, a single-center study limited to one university hospital, and the lack of mRS assessment data 90 days post-stroke.

Although LA is not fully understood, it is clearly an important and independent prognostic factor for both functional outcome and mortality among ischemic stroke and ICH patients. Thus, LA seems to be an important target for treatment intervention. Future research should aim to understand the pathophysiology of LA to improve stroke prevention.

## Data Availability

The raw data supporting the conclusions of this article will be made available by the authors upon reasonable request.
